# Tensile deformation mechanisms of an *in-situ* Ti-based metallic glass matrix composite at cryogenic temperature

**DOI:** 10.1038/srep32287

**Published:** 2016-08-31

**Authors:** J. Bai, J. S. Li, J. W. Qiao, J. Wang, R. Feng, H. C. Kou, P. K. Liaw

**Affiliations:** 1 State Key Laboratory of Solidification Processing, Northwestern Polytechnical University, Xi’an 710072, China; 2 Department of Materials Science and Engineering, University of Tennessee, Knoxville, TN 37996-2200, USA; 3 Laboratory of Applied Physics and Mechanics of Advanced Materials, Taiyuan University of Technology, Taiyuan 030024, China

## Abstract

Remarkable tensile ductility was first obtained in an *in-situ* Ti-based bulk metallic glass (BMG) composite at cryogenic temperature (77 K). The novel cryogenic tensile plasticity is related to the effective accommodation of ductile body-centered cubic dendrites at 77 K, characteristic of the prevailing slip bands and dislocations, as well as lattice disorder, which can effectively hinder the propagation of critical shear bands. The greatly increased yield strength of dendrites contributes to the high yield strength of composite at 77 K. A trend of stronger softening is observed at low temperature, and a criterion is proposed to understand the softening behavior. The current research could also provide a guidance to the promising cryogenic application of these new advanced BMG composites.

Alleviating the catastrophic failure in bulk metallic glasses (BMGs) caused by the highly-localized shear banding deformation is still full of challenges^1^. The advent of BMG composites with *in-situ* precipitated crystalline dendrites has provided an effective and promising way to circumvent the brittleness^2^,^3^,^4^,^5^,^6^,^7^. By means of the good coordination between the crystalline dendrites and glass matrix, enhanced ambient plasticity and high strength have been obtained simultaneously, which also render them as promising candidates of advanced engineering structural materials^8^,^9^,^10^.

The ambient mechanical properties of these *in-situ* BMG composites under quasi-static deformation have been extensively studied^11^,^12^. However, up to now, deformation behaviors under many kinds of extreme conditions, such as cryogenic temperature^13^, are still barely investigated. With the ever increasing demand of advanced materials for extending their applications, the exploration of deformation behaviors to some extreme conditions other than ambient or quasi-static condition is becoming more and more important. For aeronautical applications, it’s useful and necessary to investigate their performance at cryogenic temperatures. In this regard, studies on the cryogenic properties of these *in-situ* BMG composites will be not only beneficial to understanding the underlying mechanisms, but also in favor of extending their potential applications and designing new BMG composites for cryogenic applications. In the present study, it’s the first time that a remarkable tensile plasticity was obtained in *in-situ* Ti-based BMG composites at cryogenic temperature. The detailed micromechanisms are explored via the synchrotron X-ray diffraction and high-resolution transmission electron microscopy (TEM).

## Methods

Master alloys of Ti_48_Zr_20_Nb_12_Cu_5_Be_18_ were produced by arc melting the mixture of pure elements, Ti, Zr, Nb, Cu > 99.99% wt. %, and Be > 99% wt.% purity. Plate-shape samples with 2 mm in thickness and 10 mm in width were obtained by the copper mould casting method. Dog-bone-like plate specimens with a gauge dimension of 15 mm (length) × 2.5 mm (width) × 1.5 mm (thickness) were well polished and used for tensile tests. Cryogenic mechanical tests were conducted on an MTS SANS CMT5105 universal testing machine with a cryogenic box under strain rate of 5 × 10^−4^ s^−1^ at 77 K by adding liquid nitrogen, and the sample temperature is monitored using PT–100 thermo resistance. The as-casted and fractured samples were observed by scanning-electronic microscopy (SEM, VEGA3 TESCAN) to examine the microstructure and fractographs. Synchrotron X-ray diffraction was performed in the beamline, 11ID-C (115 keV), of the Advanced Photon Source, Argonne National Laboratory, IL. The deformed samples after tension were studied by TEM with high resolution in Tecnai G2 F30 to identify the micromechanism.

## Results

**Figure 1:**
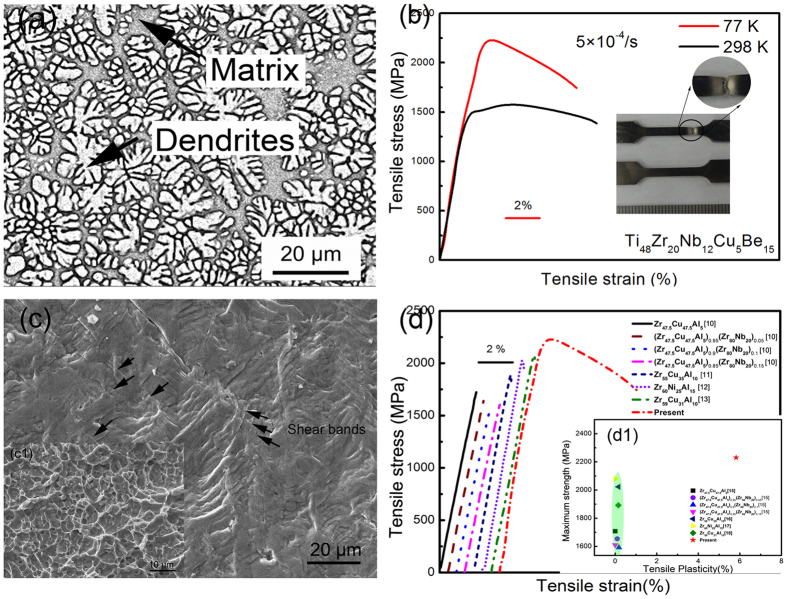
(**a**) The as-casted microstructure of the present BMG composite (**b**) Tensile stress vs. strain curves of the present Ti-based BMG composite at 77 K and 298 K. The inset shows the necking of the sample after deformation, corresponding to the remarkable plasticity of the present BMG composites at 77 K. (**c**) the lateral surface after tensile deformation at 77 K, demonstrating the multiplication of shear bands. (c1) Dimple pattern after fracture. (**d**) and (d1) The comparison of tensile properties of the present BMG composite with BMGs and composites reported before at cryogenic temperature, showing that the present Ti-based BMG composite owns novel cryogenic tensile properties.

Figure 1(a) exhibits the as-casted microstructure of the present BMG composites. It can be seen that *in-situ* precipitated dendritic secondary phase is homogenously embedded in the continuous matrix. The volume fraction of the dendrites is estimated to be approximately 52%, and the spacing of primary dendrites is about 1–3 μm. Figure 1(b) exhibits the tensile engineering stress vs. strain curve of the present BMG composites under the strain rate of 5 × 10^−4^ /s at 77 K. Up to now, only ambient and high-temperature tensile plasticity are reported in BMG composites^4^,^14^. It’s worth noting that a novel tensile plasticity of 8% and yield strength of 2,120 MPa were first obtained in the present BMG composites at cryogenic temperature. For reference, the ambient tensile stress-strain curve of the present BMG composite is also exhibited in Fig. 1(b). The present BMG composite has greatly increased yield strength at 77 K. Compared with the conventional crystalline Ti-alloys materials for cryogenic applications, the present BMG composite owns much higher fracture strength of 2,230 MPa than the pure-Ti with the average fracture strength of 1,100 MPa and Ti-6Al-4V of 1,300–1,500 MPa^15^ at 77 K, which reveals that BMG composites could be promising materials for cryogenic applications. As shown in the inset of Fig. 1(b), an obvious necking can be observed, corresponding to the large tensile softening plasticity. Figure 1(c) is the SEM image of the lateral surface of the fracture sample. Very dense shear bands with a space of 1–2 μm indicate the multiplication of shear bands at 77 K. Meanwhile, after fracture, dimple patterns, as a characteristic of ductile fracture, can be observed in the inset (c1). Figure 1(d) summaries the cryogenic tensile properties of the monolithic BMGs and *in-situ* dendrite-reinforced BMG composites^16^,^17^,^18^,^19^. Brittle tensile fracture is widely observed in both BMGs and composites reported before at 77 K, as shown in the inset (d1). In monolithic BMGs, once the critical shear band initials in the matrix under tensile stress, it will mature rapidly to generate cracks for lack of effective obstacles to hinder the rapid propagation of shear bands^20^.

**Figure 2:**
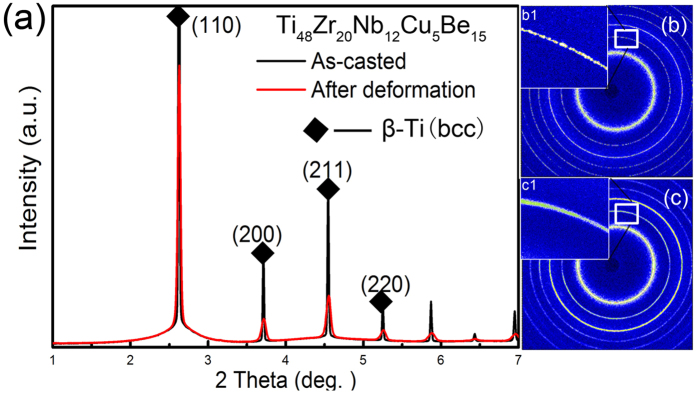
(**a**) Synchrotron X-ray line profiles of the present BMG composite before and after deformation. (**b**,**c**) the typical diffraction patterns before (**b**) and after (**c**) deformation. Inset (b1) and (c1) are the magnified rectangular regions in (**b**,**c**), respectively, indicating the fragmentation inside dendrites.

To clarify the deformation mechanisms of the present BMG composites, synchrotron X-ray diffractions were conducted on the undeformed and cryogenic deformed samples, as displayed in Fig. 2. In contrast to the undeformed sample, the full width at half maximum (FWHM) of the β-Ti dendrite peaks becomes widen and the peak intensities decrease after deformation, as shown in Fig. 2(a). Qiao *et al*.^21^ and Ott *et al*.^22^ have indicated that the peak intensity of the amorphous matrix is much weaker than FWHM of crystalline diffraction peaks. Thus, the larger FWHM represents the micro-deformation in dendrites, indicative of an obvious refinement of the dendrites^11^. Figure 2(b,c) are the typical diffraction patterns of the undeformed and deformed samples, respectively. As displayed in the magnified rectangular region of Fig. 2(b), the discrete diffraction spots correspond to the diffraction of micro-level β-Ti dendrites with different crystalline orientations. Comparing Fig. 2(b1,c1), the discrete diffraction rings of β-Ti dendrites turn to be continuous after tension, which implies the severe fragmentation inside dendrites^11^. Therefore, at cryogenic temperature, the fragment of dendrites plays an important role on hindering the highly localized shear-banding deformation and stimulating the formation of multiple shear bands^21^. Meanwhile, no additional crystalline peaks are detected after deformation at 77 K, indicating that no phase transformation occurs.

**Figure 3:**
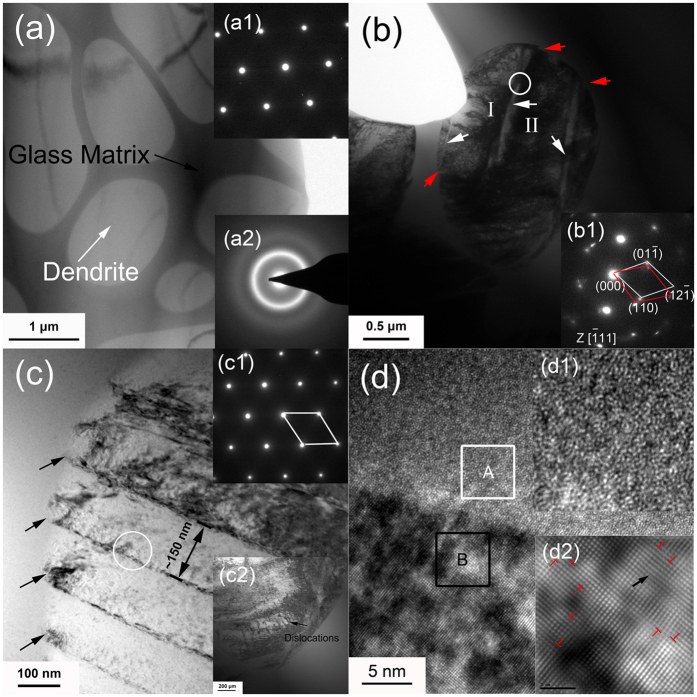
(**a**) Bright-field TEM image of the undeformed microstructure of the present BMG composite. The inset (a1) and (a2) are the SAED patterns of the dendrites and glass matrix, respectively. (**b**) Bright-field TEM image of the dendrites after deformation. The inset (b1) is the SAED pattern of the region marked by the white circle, indicating the interaction between the shear bands and dendrites. (**c**) The magnified TEM image inside the dendrites, demonstrating the prevailing of slip bands in the dendrites after deformation. The inset (c1) and (c2) showing SAED pattern of the region denoted by white circle in (**c**), and dense dislocations in dendrites, respectively. (**d**) The HRTEM image of the interface between the dendrite and glass matrix. (d1) and (d2) giving IFFT images of the region A and B, respectively.

Figure 3(a) is the bright-field TEM image of the undeformed sample. The dendrites (white contrast) are homogenously embedded in the continuous matrix (dark contrast). The inset (a1) and (a2) are the corresponding SAED patterns of the dendrites with a zone axes of [−111] and glass matrix, further confirming the amorphous structure of matrix. As shown in Fig. 3(b), after cryogenic deformation, some bands (marked by white arrows) with a space about 0.5–1 um can be observed which have the same order of magnitude to the space of shear bands formed on the lateral surface [Fig. 1(b)]. To identify these bands, the SAED pattern of the region marked by the white circle is shown in the inset of Fig. 3(b). Although the zone axes are still identified as the [−111] for both sides of dendrites with a lattice of 0.3243 nm, the rotation of diffraction patterns reveals the relative movement of two subdivisions, I and II, indicating the propagation of shear bands inside the dendrites. It can also be observed that some bands were caught and stopped propagation in dendrites, which demonstrates that the rapid propagation of shear bands has been effectively retarded. Shear steps (denoted by red arrows) form on the interface between dendrites and glass matrix. In the magnified image of dendrites [Fig. 3(c)], parallel bands with an average span of ~150 nm are prevalent, which is in accordance with previous results in BMG composites and nanostructure materials at ambient temperature^23^,^24^. The similar SAED patterns on both sides of the bands in the inset (c1) further demonstrate that they are slip bands move along the same special direction in the dendrites. As marked by black arrows in Fig. 3(c), a stepped morphology forms at the interfaces caused by the accumulation of dislocations during sliding of slip bands^11^,^23^. Corresponding to the severe plastic deformation in dendrites, dense dislocations generate at the same time in the inset (c2). Fig. 3(d) exhibits the high resolution TEM image of the interface between the dendrite and matrix, indicating the good atomic bonding between the matrix and dendrites, which also ensures good load transfer during deformation. The corresponding inverse fast Fourier transformed (IFFT) images of the rectangular region, A and B, are displayed in Fig. 3(d1,d2). Maze-like atomic arrangement in the inset (d1) further reveals that no nanocrystalization occurs in the matrix. Multiple dislocations denoted by “T” and the lattice distortion marked by the black arrow can be observed in the inset (d2), which indicates the severe stress concentration in dendrites during deformation. It has been reported that in BCC metals and alloys, such as pure Fe, due to dramatically increased resistance to the dislocation slip at cryogenic temperatures, deformation twinning becomes a favorable deformation mode^25^. However, the above results demonstrate the absence of deformation twinning at cryogenic temperature. Thus, as far as the present BMG composite, the effective accommodation of dendrites at cryogenic temperature, characteristic of the formation of shear steps, multiple dislocations and slip bands, has greatly favored the remarkable macroscopic plasticity by hindering the propagation of shear bands.

## Discussion

For BMG composites, some deformation mechanisms have been proposed to illustrate the effects of secondary phases at ambient temperature. For example, to some Ti-26 and CuZr-based^27^ BMG composites, the deformation-induced “phase transformation” in the secondary phase plays an important role in the accommodation of the plastic strain and the multiplication of shear bands by tuning shear-stress distribution. On the other hand, for some dendrite-reinforced Ti-, and Zr-based BMG composites, which consist of the “soft” second phase with low shear modulus, the dendrites could effectively consume the shearing energy by retarding and arresting the propagation of shear bands, i.e., “absorption effect”^4^,^24^. However, at 77 K, brittle facture has been widely reported previously in these *in-situ* Ti- and Zr-based dendrite-reinforced BMG composites even under compression^13^,^28^. The main reason lies on the ductile to brittle transition of body-centered cubic (BCC) dendrites at low temperature, which leads to the formation of premature cracks. However, for the present BMG composites, the intense slip bands and dislocations inside dendrites confirm that the dendrites still own favorable ability to accommodate the glass matrix at least at 77 K. In addition, although decreased toughness of the dendrites is still expected at low temperature for the present BMG composites, it’s been documented that the plasticity of BMGs will increase at low temperatures because of the retarded shear velocity^29^,^30^, enhanced atomic disorder^31^ etc. Thus, a more ductile glass matrix at low temperature should also be in favor of relieving deteriorated “absorbing” effects of dendrites and resulting in ameliorative macroscopic plasticity for the present BMG composites. Meanwhile, in comparison to what happened at ambient temperature, the yield strength increase approximately 49% at 77 K for the present composite. Fan *et al*.^32^ claimed that the increased compressive yield strength in the Ta-particle reinforced Zr-based BMG composite at 77 K should be attributed to the reduced thermal vibrations of the glass matrix. Qiao *et al*.^13^ further found that the increase of yield strength from 298 K to 77 K has a range of 314 MPa ± 80 MPa, which is much lower than the increased yield strength of about 700 MPa in the present composite at 77 K, implying the non-ignorable effect of dendrites on yielding. Compared with the glass matrix, the dendrites with a BCC structure own more pronounced temperature dependence due to the greatly increased dislocation friction at low temperature^32^, which can be expressed as *σ*_*friction*_ = *Bexp*(*−CT*) (*B* and *C* are positive constants)^33^. Previous studies^34^ have claimed the yield strength of crystalline β-Ti alloys can increase up to twice at 77 K. In *in-situ* dendrite-reinforced BMG composites At cryogenic temperatures, the dendrites are still supposed to yield prior to the glass matrix at cryogenic temperature^13^. Thus, it’s reasonable to deduce that the dendrites dominate the high yield strength of the present BMG composite at 77 K, which is different from the Ta-particle reinforced BMG composite during compression^32^.

**Figure 4:**
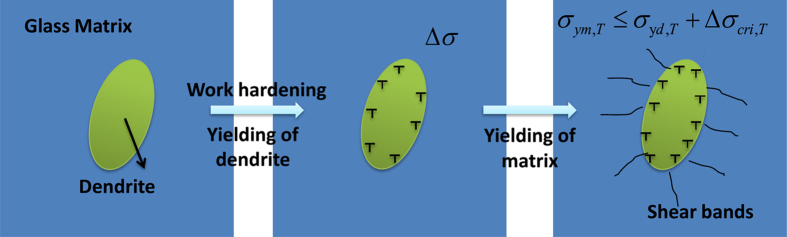
The schematic of the deformation behavior in the present BMG composites.

Moreover, note that compared with that at 298 K in Fig. 1(b), the work-hardening stage at 77 K is dramatically reduced and displays a stronger downward trend before the final fracture, revealing the subdued work-hardening ability. After yielding, the deformation of BMG composites could be divided into two stages as shown in Fig. 4. First, the severe deformation in the dendrites causes the formation and accumulation of dense dislocations, which results in the strain hardening of the composites. Then, once the critical stress concentration due to strain hardening in dendrites reaches the yielding strength of the glass matrix, multiple shear bands initials in the glass matrix, which leads to the rapid softening of the glass matrix and the stress relaxation^5^,^11^. It has been demonstrated that the work-hardening ability of β dendrites exhibits negligible variation from 298 K to 77 K^15^. Thus, the more vulnerable to initiate shear bands in the glass matrix, the stronger trend of softening. The critical stress for the yield of the glass matrix can be given as,


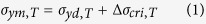


where, Δ*σ*_*cri,T*_ and *σ*_*yd*,*T*_ are the critical stress concentration needed to initiate the yield of the glass matrix and the yield strength of dendrites at a certain temperature, *T*, respectively. Then, the difference between critical stresses concentration needed to initiate the shear bands in the glass matrix at 77 K and 298 K is obtained,


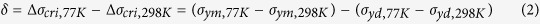


During deformation, the dendrites are assumed to yield first^11^,^13^. The yield strength of dendrites can be given as *σ*_*yc*_ = *c*_*d*_ *σ*_*yd*_, where *c*_*d*_ is the stress concentration factor, and *c*_*d*_ ≈ 1 for the *in-situ* BMG composites^11^,^35^. Then, Eq. 2 can be roughly transformed as,


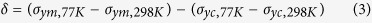


Equation (3) gives a simple criterion to determine whether the trend of softening will be more pronounced at cryogenic temperature. If *δ* < 0, i.e., △*σ*_*cri,77K*_ < Δ*σ*_*cri,298K*_, which implies that at 77 K, a lower level of hardening in dendrites is needed before reaching the yielding point of the glass matrix. In other words, during deformation, shear bands are easier to initiate in the matrix, causing stronger softening at low temperatures. Here, the average 314 MPa^13^ of *σ*_*ym,77K*_ − *σ*_*ym,298K*_ is taken for the glass matrix. The yield strengths of the present BMG composite at 298 K and 77 K are determined to be 1,420 MPa and 2,120 MPa respectively. Then, the difference of critical stresses at 298 K and 77 K can be obtained, *δ* = −426 MPa < 0 for the present BMG composite. Thus, compared with the ambient temperature, the present BMG composite exhibits a subdued strain hardening at 77 K. It can also help understand why Ta particle-reinforced Zr-based BMG composites do not exhibit stronger softening at 77 K^32^, but stronger softening is observed in some dendrite-reinforced Ti-based BMG composites^28^, where *δ* can be estimated to be 34MPa > 0 and –236 MPa < 0, respectively. Therefore, dendrite-reinforced Ti-based BMG composites exhibit obvious strain softening at lower temperature^28^.

Finally, as discussed above, the ductile dendrites at cryogenic temperature, characteristic of the intense slip bands in the dendrites, as well as multiplication of dislocation and lattice disordered, is vital to hinder the rapid propagation of shear bands and accommodate the deformation of the glass matrix. This could also provide a guidance to design new BMG composites with great low-temperature properties.

## Conclusion

In summary, the tensile deformation behavior of an *in-situ* Ti-based BMG composite was investigated at 77 K. It’s the first time that a novel cryogenic tensile plasticity (8%), together with high fracture strength of 2,230 MPa was obtained at cryogenic temperature. The ductile dendrites even at 77 K, characteristic of the prevailing slip bands and dislocations, as well as lattice disorder contribute to the remarkable tensile plasticity by arresting the rapid propagation of individual shear bands and accommodating plastic deformation. The high yield strength of the present BMG composites can be attributed to the greatly increased yield strength of dendrites at 77 K. A simple criterion is proposed to understand the softening trend of BMG composites at low temperature.

## Additional Information

**How to cite this article**: Bai, J. *et al*. Tensile deformation mechanisms of an *in-situ* Ti-based metallic glass matrix composite at cryogenic temperature. *Sci. Rep.* **6**, 32287; doi: 10.1038/srep32287 (2016).
